# Boron and Silicon-Substituted 1,3-Dienes and Dienophiles and Their Use in Diels-Alder Reactions

**DOI:** 10.3390/molecules25163740

**Published:** 2020-08-16

**Authors:** Mark E. Welker

**Affiliations:** Department of Chemistry, Center for Functional Materials, Wake Forest University, 455 Vine Street, Winston-Salem, NC 27101, USA; welker@wfu.edu; Tel.: +1-336-702-1953

**Keywords:** boron, silicon, Diels-Alder, diene, dienophile

## Abstract

Boron and silicon-substituted 1,3-dienes and boron and silicon-substituted alkenes and alkynes have been known for years and the last 10 years have seen a number of new reports of their preparation and use in Diels-Alder reactions. This review first covers boron-substituted dienes and dienophiles and then moves on to discuss silicon-substituted dienes and dienophiles.

## 1. Introduction

This review article covers recent reports of boron and silicon-substituted 1,3-dienes and dienophiles and their use in Diels-Alder reactions. Reviews on boron diene/Diels-Alder chemistry were published in 2017 [1] and 2014 [2] so this review will cover that topic from 2016–2020. Silyl-substituted 1,3-dienes and their Diels-Alder reactions were reviewed in early 2011 [3] so this review will cover that chemistry from 2010–2020. The articles reviewed here came from one of four topical searches within the Science Citation Index. A search using keywords boron and diene between 2016 and 2020 yielded 51 references, which were subsequently checked for reported organic chemistry. A second search over the same period using keywords boron and Diels-Alder yielded 76 references. Similar searches over 2010–2020 using silicon and diene and silicon and Diels-Alder yielded 101 and 137 references respectively, which were all checked for diene preparation and/or reports of Diels-Alder chemistry. In terms of review format, boron and silicon dienes and dienophiles and their Diels-Alder reactions will be presented chronologically from oldest to most recent reports within the topical areas of boron dienes, boron dienophiles, silicon dienes, and silicon dienophiles.

## 2. Boron Dienes

### 2.1. Boron Dienes Other Than Boroles

**Scheme 1:**
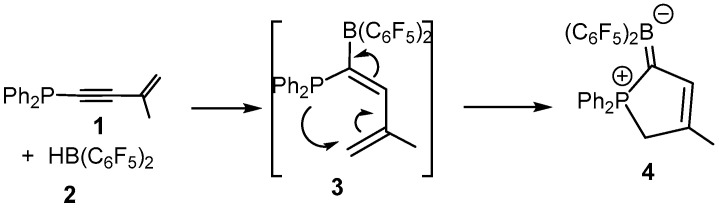
Preparation of borata-dienes.

Erker and co-workers reported hydroboration of a phosphorus-substituted enyne (**1**) to produce a proposed boron-substituted 1,3-diene (**3**) which presumably cyclized to the isolated borata-dienes (**4**) (Scheme 1) [4]. While this chemistry has a proposed boron diene intermediate (**3**), the intramolecular diene cyclization proposed presumably precludes any diene trapping of this intermediate via Diels-Alder chemistry, but furthermore, running the reaction in the presence of a dienophile would provide that answer.

**Scheme 2:**
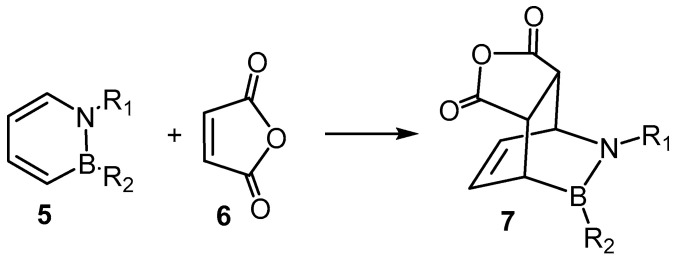
Diels-Alder reactions of azaborines.

Fernandez and co-workers reported a computational study on the factors that control *endo*/*exo* selectivity of Diels-Alder reactions of 1,2-azaborines (**5**) [5] which had been reported experimentally in 2015 (Scheme 2) [6]. Their computations predict/rationalize endo products (**7**) but the main reason for lack of exo selectivity was predicted to be due to the higher deformation energy for the exo pathway.

**Scheme 3:**
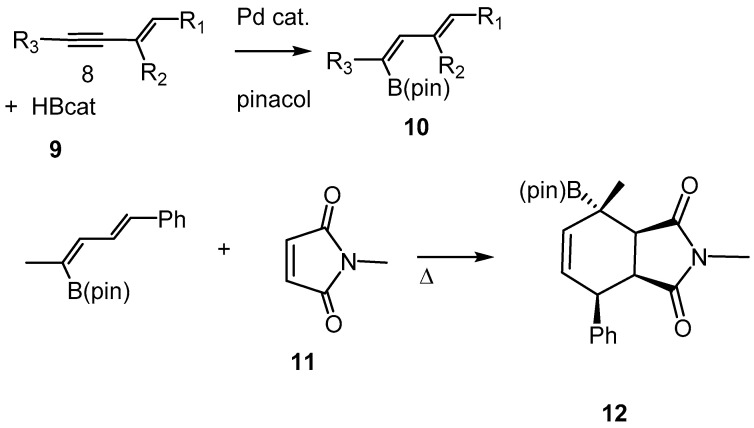
Hydroboration of 1,3-enynes.

Liu and co-workers reported a *trans*-hydroboration of 1,3-enynes (**8**) to produce 1-boron-substituted-1,3-dienes (**10**) (Scheme 3) [7]. This unusual *trans* hydroboration was enabled by a 1,4-azaborine-based phosphine-Pd complex catalyst, which is specific for *trans* hydroboration. The hydroboration worked for both terminal and internal alkynes and in one instance, the diene produced was used in a Diels-Alder reaction with *N*-methylmaleimide (**11**).

**Scheme 4:**
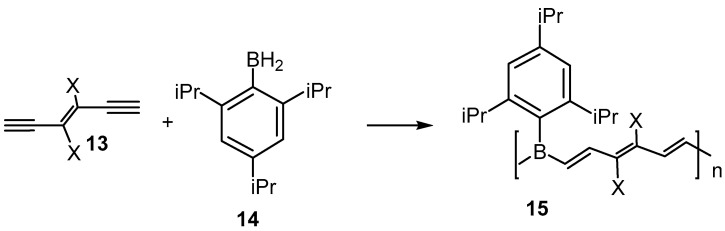
Hydroboration of yne-ene-ynes.

Qin published an account of his group’s work making boron doped polyacetylenes (BDPA) via hydroboration of yne-ene-ynes (**13**) (Scheme 4) [8]. The products of this reaction (**15**) contain boron-substituted diene or triene fragments so this work is included here for completeness.

**Scheme 5:**
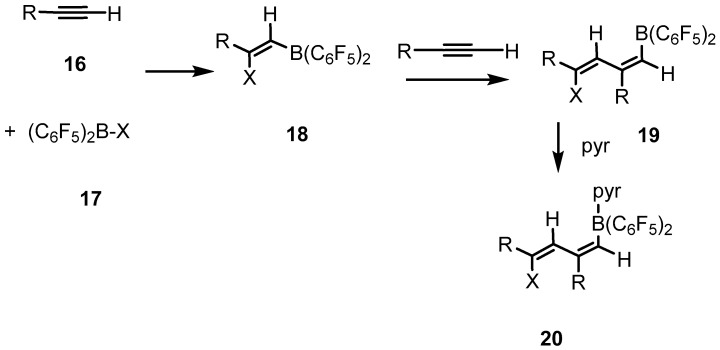
Preparation of (C_6_F_5_)_2_B-substituted dienes.

Erker and co-workers reported reactions of (C_6_F_5_)_2_BX (**17**) with terminal alkynes (**16**) to generate 1-(C_6_F_5_)_2_B-substituted 1,3-dienes (**19**) (Scheme 5) [9]. The reaction sequence used to generate the products: 1,2-halogenoboration of an alkyne, followed by 1,2-carboboration of a second equivalent of that alkyne, is unusual. Addition of pyridine to the reaction mixture allowed isolation of the boron dienes (**20**) and in the absence of a pyridine, alkyne oligomerization occurred.

**Scheme 6:**
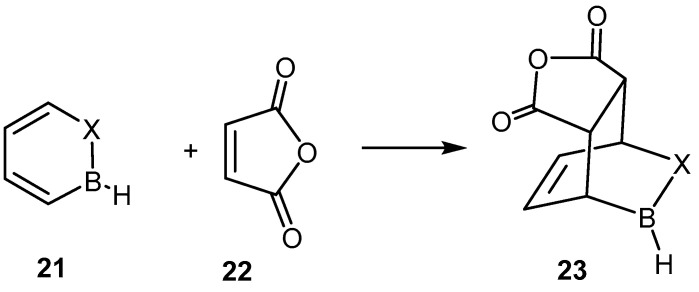
Diels-Alder reactions of borines.

Fernandez and co-workers reported a follow up computational study of Diels-Alder reactivity of a number of 1-other element-2-borine analogs (**21**) with maleic anhydride (**22**) (Scheme 6) [10]. As mentioned above, they had done this kind of work for azaborines earlier and here they concluded that Diels-Alder reactions should become more favorable from left to right in a period (X = C < N < O) and when going down in a group (X = N < P < As).

**Scheme 7:**
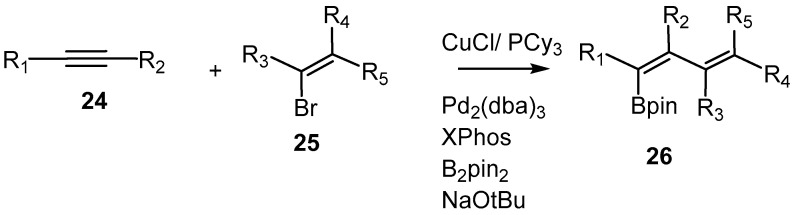
Preparation of 1-boron-substituted dienes.

Fananas-Mastral and Vazquez-Galinanes reported synergistic Cu/Pd-catalyzed couplings of alkynes (**24**) and bromoalkenes (**25**) to stereoselectively make 1-boron-substituted-1,3-dienes (**26**) (Scheme 7) [11]. In one case, a boron diene thus produced was cross-coupled to produce a triene but no Diels-Alder chemistry of these dienes has yet been reported.

**Scheme 8:**
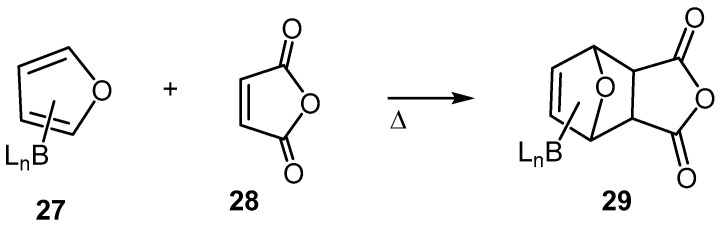
Diels-Alder reactions of boron-substituted furans.

Lastly in recent boron diene chemistry, Pellegrinet and co-workers reported preparation of 2- and 3-boron-substituted furans (**27**) and found that while the 3-boron-substituted furans reacted well with maleic anhydride (**28**), the 2-boron-substituted furans failed to do so (Scheme 8) [12]. Theoretical calculations on these two types of reactions were also performed and the failure of the 2-boron-substituted dienes to react was largely attributed to steric distortion experienced with the dienophile when boron was in the 2 position.

### 2.2. Boroles

Boroles are anti-aromatic heterocycles that one can consider as a special kind of boron-substituted diene, and they continue to be an active area of research. They are broken out here into their own separate category within boron diene chemistry.

**Scheme 9:**
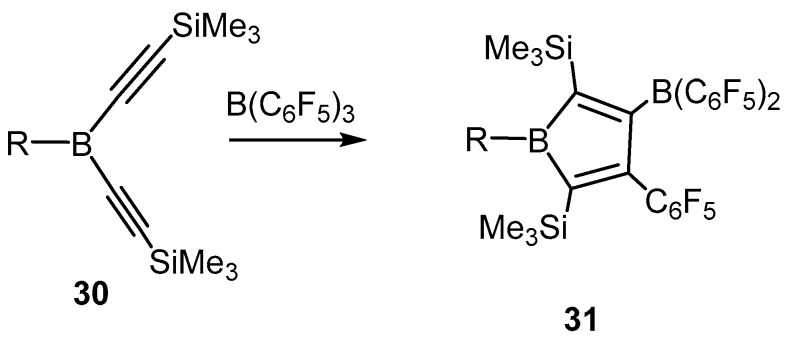
Carboboration of diynes.

Wrackmeyer and Khan have worked in this area for a number of years and authored a microreview that appeared in 2016 (Scheme 9) [13]. The synthetic chemistry covered involves 1,1-carboboration of diynes (**30**) to yield boroles (**31**), siloles, and stannoles. The general reaction used to make boroles is shown below.

**Scheme 10:**
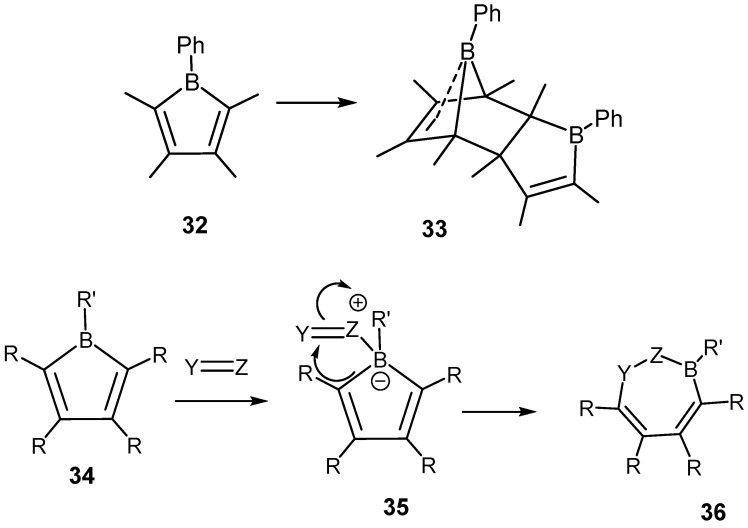
Diels-Alder and ring expansion of boroles.

Martin and co-workers also published a review on boroles in 2016 but this review covers Diels-Alder and ring expansion reactions of boroles (Scheme 10) [14]. In general, in the absence of Lewis bases, boroles **32** dimerize to **33** or react with other potential dienophiles via Diels-Alder chemistry and in the presence of Lewis bases boroles generally undergo ring expansion **36**.

**Scheme 11:**
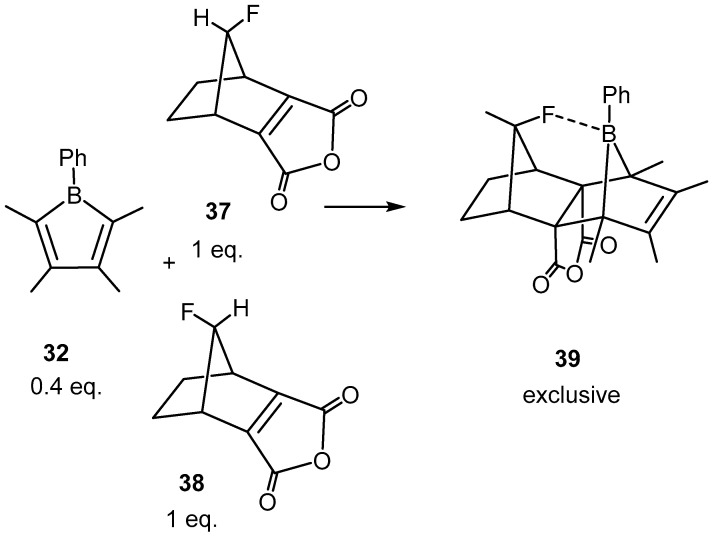
C-F bond directed Diels-Alder reaction.

Lectka and co-workers reported both an experimental and theoretical analysis of a C-F bond directed Diels-Alder reaction of pentasubstituted boroles (Scheme 11) [15]. The pivotal experimental result was a competitive Diels-Alder reaction between phenyltetramethylborole (**32**) and the syn and anti fluoro isomers of a methanoisobenzofuran 1,3-dione (**37** and **38)**. The B-F coordinated Diels-Alder adduct (**39**) was the exclusive product.

**Scheme 12:**
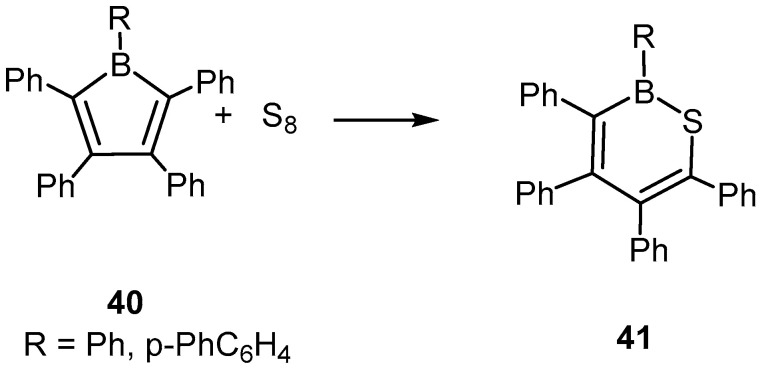
Preparation of thiaborines.

Martin and Yruegas reported the ring expansion reaction of boroles outlined above in Scheme 10 as a route to 1,2-thiaborines (**41**) in 2016 (Scheme 12) [16]. Two thiaborines were reported and their UV maxima were significantly red shifted in relation to hexaphenylbenzene. Their nuclear independent chemical shift values indicated they were significantly more aromatic than the one previously reported example of this heterocycle which contained a nitrogen substituent on boron.

**Scheme 13:**
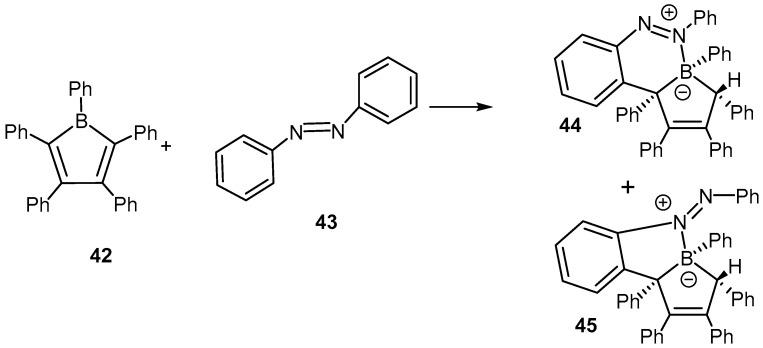
Reactions of diazenes with pentaphenylborole.

Martin and co-workers reported thermal and photochemical cyclization reactions of *cis* and *trans* diazenes (**43**) with pentaphenylborole (**42**) (Scheme 13) [17]. The cyclization products (**44** and **45**) of thermal reactions of *trans* diazenes depended on the diazene substituents. *Trans*-diphenyldiazene (**43**) gave a 2:1 mixture of two products (**44** and **45**) that differ in which nitrogen coordinates to boron. Both products were thought to arise from initial coordination of N to B followed by an intramolecular electrophilic addition of the borole diene to the carbon ortho to the coordinated nitrogen.

**Scheme 14:**
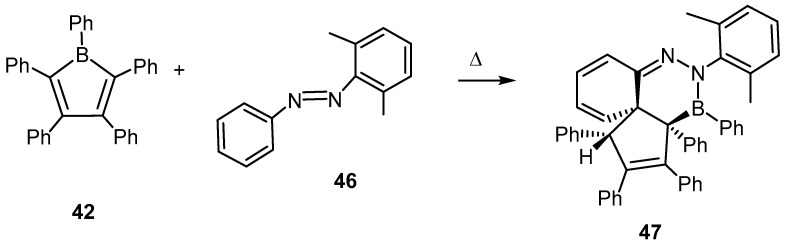
Reaction of dimethylazobenzene with pentaphenylborole.

The reaction of pentaphenylborole (**42**) with 2′,6′-dimethylazobenzene (**46**) was thought to start similarly but steric interactions between the two methyls and the phenyl groups in the analog to the major isomer (**44**) were proposed to cause a ring expansion/ring contraction sequence that led to the isolated product (**47**) (Scheme 14).

**Scheme 15:**
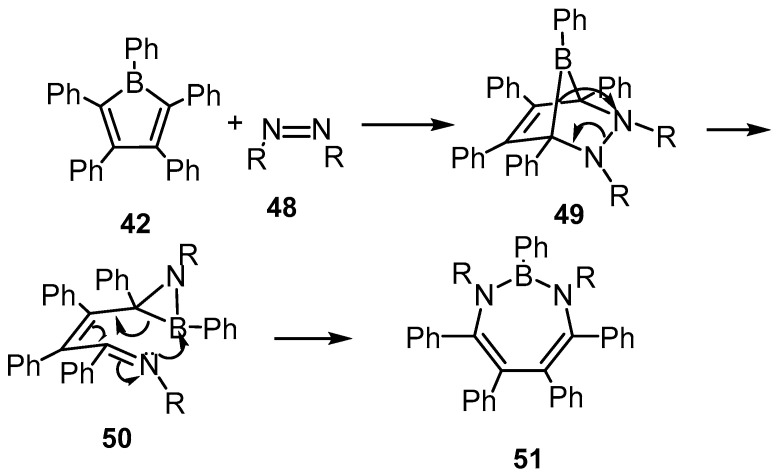
Reactions of *cis*-diazenes.

Photochemical reactions with both *trans* diazenes led to the same type of 1,3,2-diazaborepins. This same ring system was formed via thermal reactions of pentaphenyl borole with *cis* diazenes so the authors proposed that photochemical *trans*-*cis* isomerization of the *trans* diazenes prior to cyclization accounted for the observed products. Formation of these 1,3,2-diazaborepins (**51**) (Scheme 15) was thought to originate from an initial Diels-Alder reaction (**52**) followed by N-N bond heterolysis via a [1,3] sigmatropic shift. Coordination of the imine nitrogen thus liberated (**53**) to boron was proposed to set off a ring expansion that led to the observed product (**54**).

**Scheme 16:**
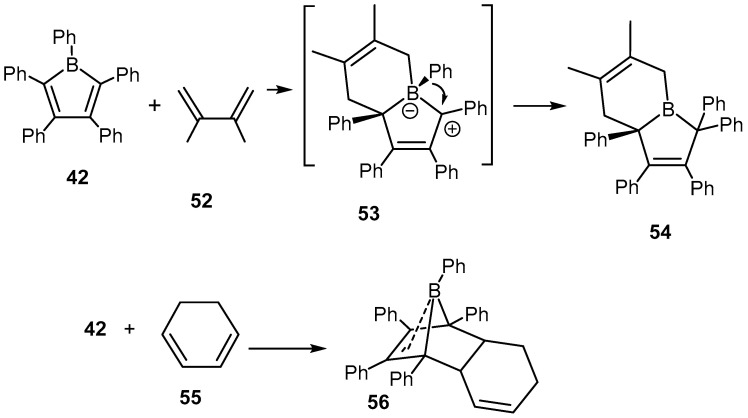
Reactions of pentaphenylborole with dienes.

Martin and co-workers continued their work and reported Diels-Alder reactions of pentaphenylborole (**42**) and the 1-phenyl-2,3,4,5-tetramethylborole dimer with 2,3-dimethyl-1,3-butadiene (**52**) and 1,3-cyclohexadiene (**55**) (Scheme 16) [18]. Pentaphenylborole (**42**) served as the dienophile in reactions with 2,3-dimethylbutadiene (**52**) whereas it functioned as the diene in [4+2] cycloaddition with cyclohexadiene (**55**).

**Scheme 17:**
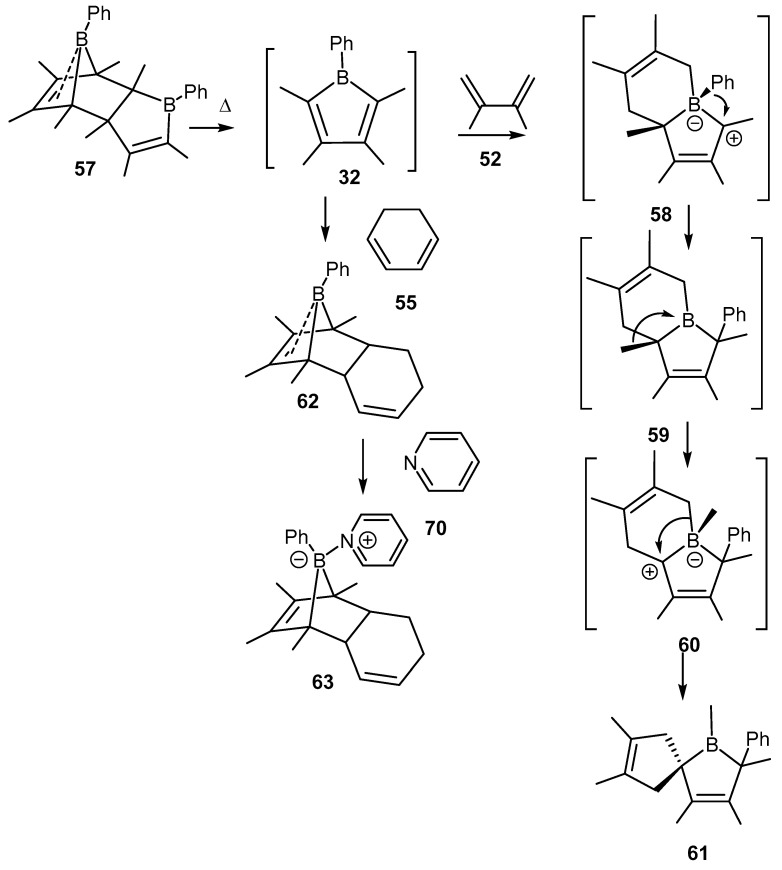
Reactions of phenyltetramethylborole dimer.

Likewise, 1-phenyl-2,3,4,5-tetramethylborole dimer (**57**) when cracked, functioned as dienophile followed by rearrangement with 2,3-dimethylbutadiene (**52**) and it functioned as diene in reaction with cyclohexadiene (**55**) (Scheme 17). Theoretical calculations indicated that steric interactions between the borole and the cyclohexadiene mainly accounted for this difference in reactivity.

**Scheme 18:**
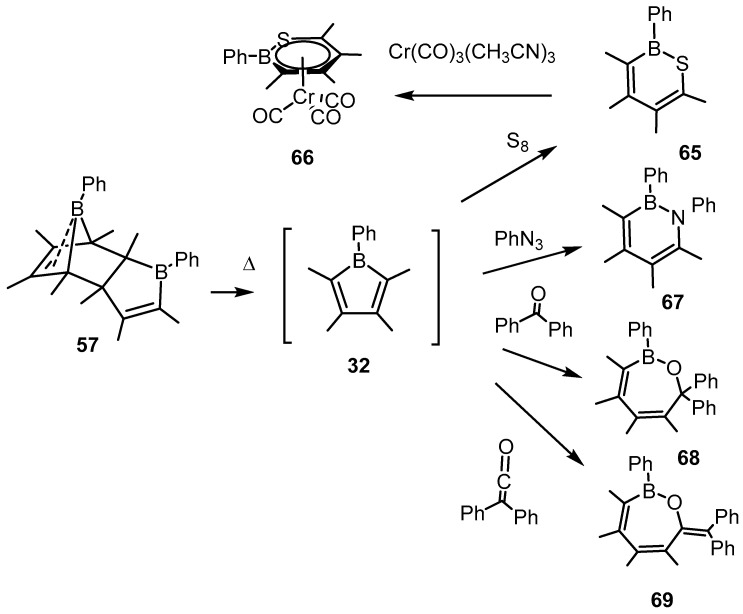
Additional reactions of phenyltetramethylborole dimer.

Martin and co-workers have continued their work on the reactions of the 1-phenyl-2,3,4,5-tetramethylborole dimer (**57**) this year (Scheme 18) [19]. Unlike pentaphenylborole, the tetramethylboroles dimerize as outlined above, and this dimer (**57**) was heated with a number of potential cycloaddition/cyclization partners in this study. Phenyl azide, sulfur, benzophenone, and diphenyl ketene all do 1,2 insertion rather than cycloaddition with this borole (**32**) presumably due to the nonbonding electrons on heteroatoms in these potential dienophiles which coordinate to boron prior to reaction.

**Scheme 19:**
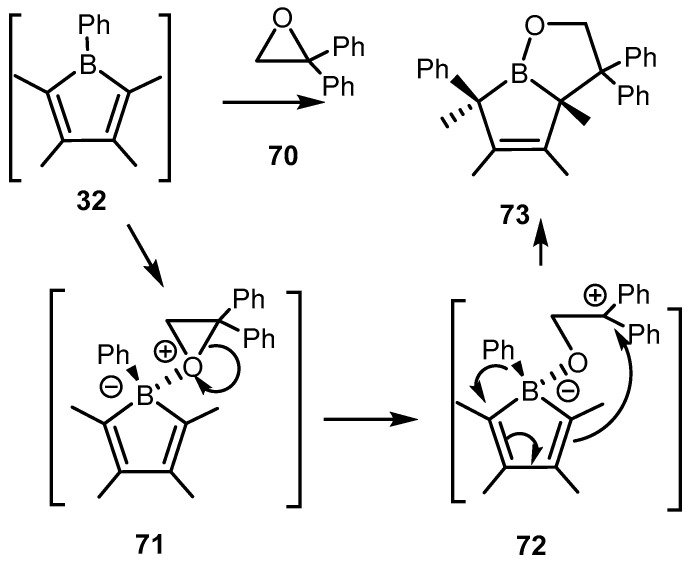
Reactions of epoxides with phenyltetramethylborole.

The epoxide, 1,1-diphenylethylene oxide (**70**), reacted with this borole (**32**) to produce an unusual fused heterocyclic compound (**73**) (Scheme 19). The mechanism proposed to account for this product began with Lewis acid mediated opening of the epoxide (**70**) to generate a highly resonance stabilized cation (**72**). Phenyl migration from that intermediate (**72**) is proposed to trigger ring closure with that cation.

**Scheme 20:**
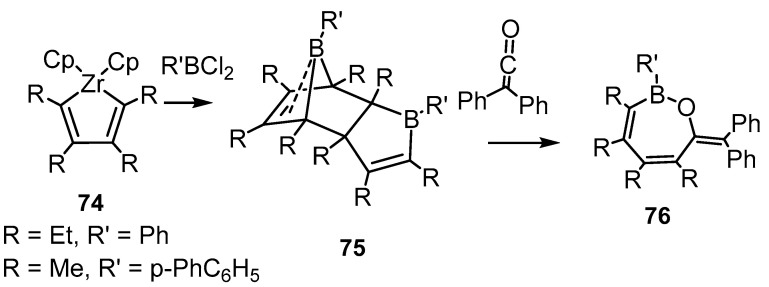
Preparation and reaction of tetraalkylborole dimers.

Lastly, they prepared two other tetraalkylborole dimers (**75**) via transmetallation of their zirconium metallocyclopentadiene precursors (**74**) (Scheme 20). The 1,2 insertion reaction of the 1-phenyl-2,3,4,5-tetraethylborole with diphenylketene was complete in one hour whereas the reaction with the larger biphenyl tetramethyl compound (**75**) required 12 h.

**Scheme 21:**
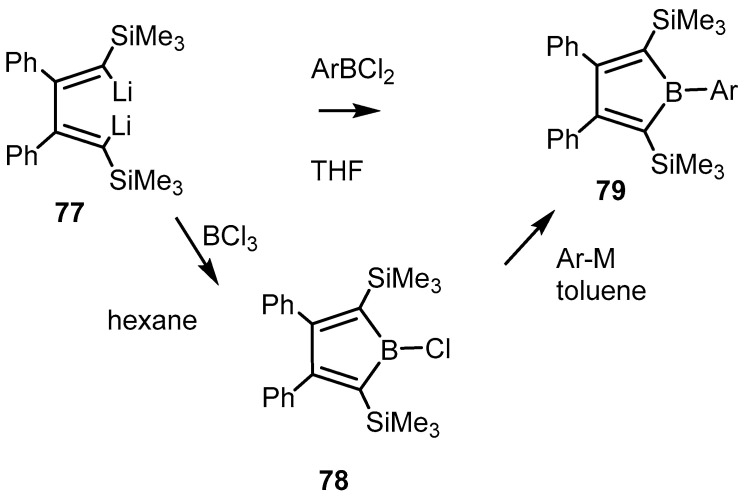
Preparation of bis-trimethylsilyl-substituted boroles.

Sindlinger and co-workers reported the preparation of several 2,5-bis-trimethylsilyl-substituted boroles (**79**) (Scheme 21) [20]. These boroles were prepared via reaction of 1,4-dilithio-1,3-butadiene (**77**) with boron electrophiles ArBCl_2_ or BCl_3_. When BCl_3_ was used as the electrophile, a chloroborole (**78**) was isolated and subsequently reacted with several aryl metal reagents in toluene.

**Scheme 22:**
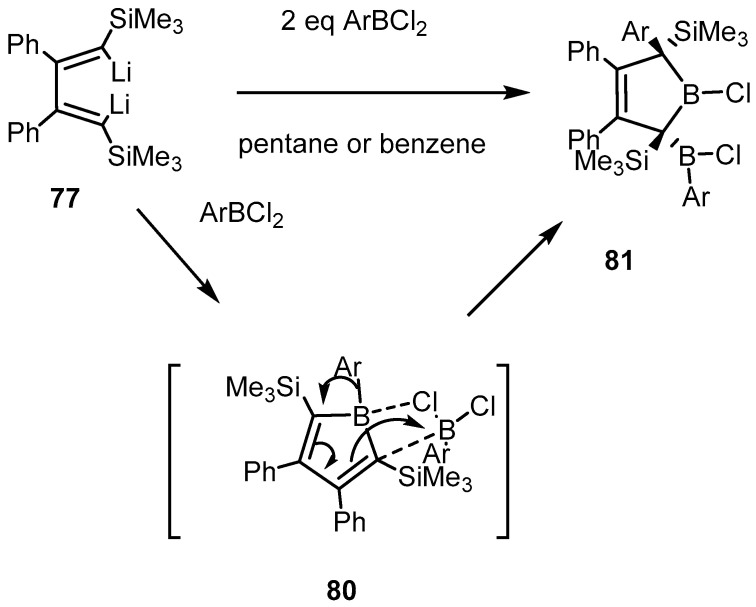
Preparation of boryl borolenes.

When the dilithiobutadiene (**77**) was treated with 2 equivalents of ArBCl_2_ in hydrocarbons, 2-boryl-3-borolenes (**81**) were isolated (Scheme 22). A proposed mechanism for this borolene formation involving Lewis acid-Lewis base interactions between the initially formed aryl borole (**80**) and ArBCl_2_ was corroborated by independently treating aryl borole (**80**) with 1 equivalent of ArBCl_2_ in hydrocarbon solvents.

## 3. Boron Dienophiles

**Scheme 23:**
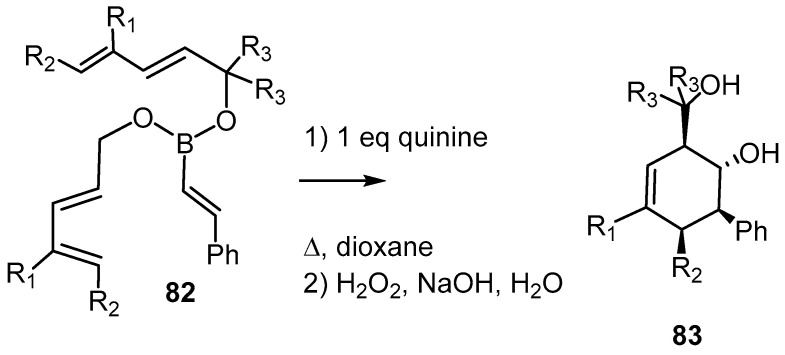
Boron tethered Diels-Alder reactions.

Several reports of boron-substituted dienophiles for use in Diels-Alder reactions have appeared in the last few years as well. In 2018, Houck, Morgan, and co-workers reported a quinine-promoted, enantioselective, boron-tethered Diels-Alder reaction of a boron-substituted dienophile (**82**) (Scheme 23) [21]. In this work, a variety of dienols were tethered to phenylethenyl boronic acid and quinine was used as a chiral promoter to coordinate to boron for the subsequent intramolecular Diels-Alder reaction. Highly substituted cyclohexenols (**83**) were recovered in good yields and enantioselectivities and quinine was recovered in good yield as well. DFT calculations were used to understand the origins of the enantioselection.

**Scheme 24:**
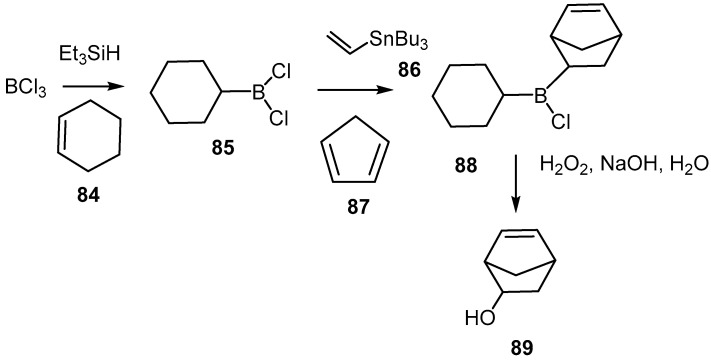
Preparation and Diels-Alder reactions of alkylhalovinyl boranes.

Pellegrinet and co-workers reported preparation of some alkylhalovinylboranes and their Diels-Alder reactions (Scheme 24) [22]. They reasoned that these vinyl boranes would be less sterically hindered and more electron deficient than dialkylvinylboranes and hence should be more reactive in Diels-Alder chemistry. In this work, they calculated activation free energies and *endo*/*exo* selectivities for a number of vinyl borane reactions with cyclopentadiene (**87**). They then tested a few of those predictions experimentally. Their proof of concept reaction involved hydroboration of cyclohexene (**84**) with dichloroborane (**85**) and Et_3_SiH followed by transmetallation with vinyltributyltin (**86**) in the presence of cyclopentadiene (**87**). Oxidation of the boron-substituted cycloadduct (**88**) produced 5-norbornen-2-ol (**89**) in 48% yield and 4:1 endo:exo selectivity.

Calculations were then performed on reactions of chiral alkylchlorovinylboranes that could be obtained by hydroboration of chiral alkenes with HBCl_2_. Diels-Alder reactions between cyclohexadiene, isoprene, and *trans*,*trans*-1,4-diphenyl-1,3-butadiene with these chiral boranes made by hydroboration of α-pinene and 2- and 3-carene were reported. Unfortunately, yields, enantioselectivities, and *endo*:*exo* selectivities of these Diels-Alder reactions were generally disappointing.

**Scheme 25:**
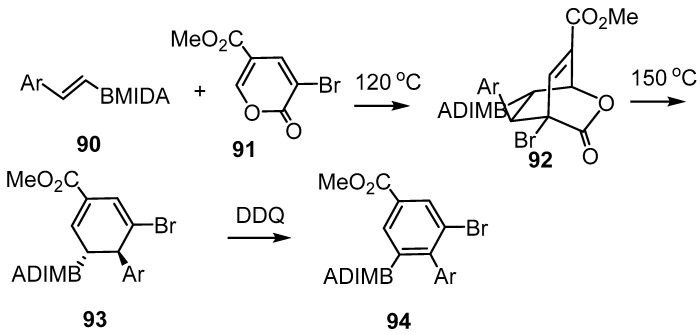
Diels-Alder reactions of alkenyl BMIDA with pyrones.

Wang and co-workers reported a number of Diels-Alder reactions of alkenyl (*N*-methyliminodiacetyl) boronates (BMIDA) (**90**) with 2-pyrones (**91**) (Scheme 25) [23]. Diels-Alder reactions of these boron dienophiles (**90**) occurred at 120 °C and decarboxylation occurred when temperatures were raised to 150 °C. If desired, the cyclohexadienyl boronates (**93**) resulting from decarboxylation could be aromatized with DDQ to produce **94** and the boron then substituted by coupling reactions or oxidation.

## 4. Silicon Dienes

### 4.1. Silicon Dienes Other Than Siloles

**Scheme 26:**
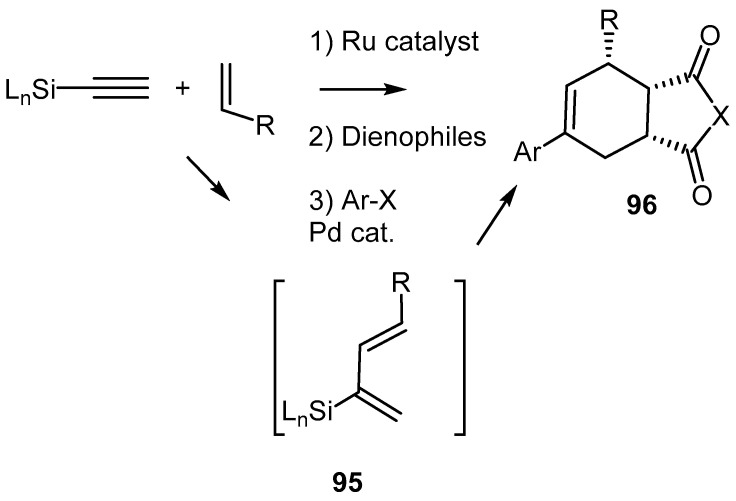
Enyne metathesis/Diels-Alder/cross coupling reactions.

In 2010, our group published an enyne metathesis route to 2-silicon-substituted 1,3-dienes (**95**) (Scheme 26) [24]. We then studied their Diels-Alder reactions and the Hiyama-Denmark cross coupling reactions (**96**) of the silicon-substituted cyclohexene products of those Diels-Alder reactions. The cross coupling allows the intermediate silicon dienes (**95**) to serve as synthons for a host of 1,3-dienes.

**Scheme 27:**
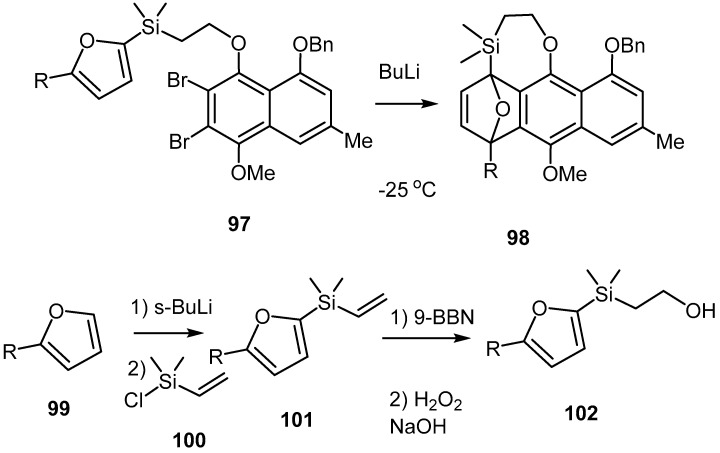
Diels-Alder reaction of a silicon-substituted furan.

Martin and co-workers communicated a total synthesis of isokidamycin that relied on a key intramolecular Diels-Alder reaction of a silicon-substituted furan (**97**) and a naphthyne (Scheme 27) [25]. This communication was followed by a full paper [26]. The silicon-substituted diene (**102**) used in this total synthesis was prepared by furan metalation followed by treatment with chlorodimethylvinylsilane (**100**) followed by hydroboration/oxidation to yield **102**.

**Scheme 28:**
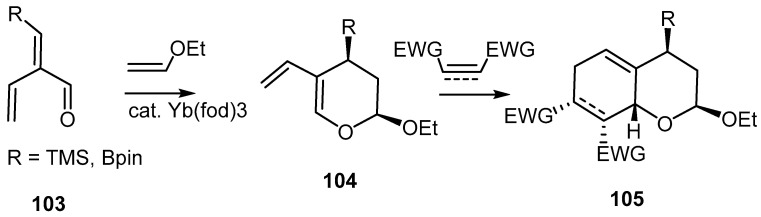
Reactions of boron and silicon-substituted dendralenes.

Carboni and co-workers reported the preparation of a silicon and a boron-substituted heterodendralene (**103**) followed by six hetero-Diels-Alder reactions of the silicon dendralene and three inverse electron demand hetero-Diels-Alder reactions of the boron dendralene (Scheme 28) [27]. The silicon and boron groups in the resulting cycloadducts (**104**) were then used in halogenation, oxidation or coupling reactions to produce more highly substituted tricyclic products (**105**).

**Scheme 29:**
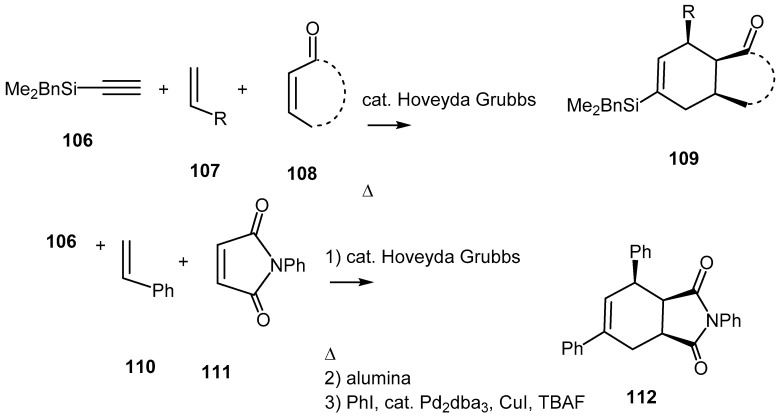
One-pot enyne metathesis/Diels-Alder reactions.

In 2012, we extended our earlier enyne metathesis work and showed that if the Hoveyda-Grubbs catalyst was used for the metathesis then enyne metathesis and Diels-Alder reactions could be run as one-pot multicomponent reactions (Scheme 29) [28]. In one case, we showed that an enyne metathesis/Diels-Alder/cross coupling sequence could be completed with only one reaction workup but addition of alumina (to chelate Ru) and its removal by filtration was required prior to cross coupling to yield **112**.

**Scheme 30:**
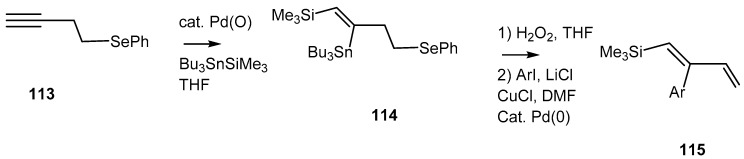
Synthesis of silyl aryl dienes.

Segi and co-workers reported a simple method for stereoselective synthesis of (*Z*)-1-silyl-2-aryl-1,3-dienes (**115**) (Scheme 30) [29]. This method used palladium-catalyzed silylstannylation of 4-(phenylselenyl)-but-1-yne (**113**) followed by oxidation-selenoxide elimination and Stille coupling. The authors found that once the initial silylstannylation was completed the oxidation-selenoxide elimination and Stille coupling could be performed in either sequence with essentially identical overall yields for the sequence.

**Scheme 31:**
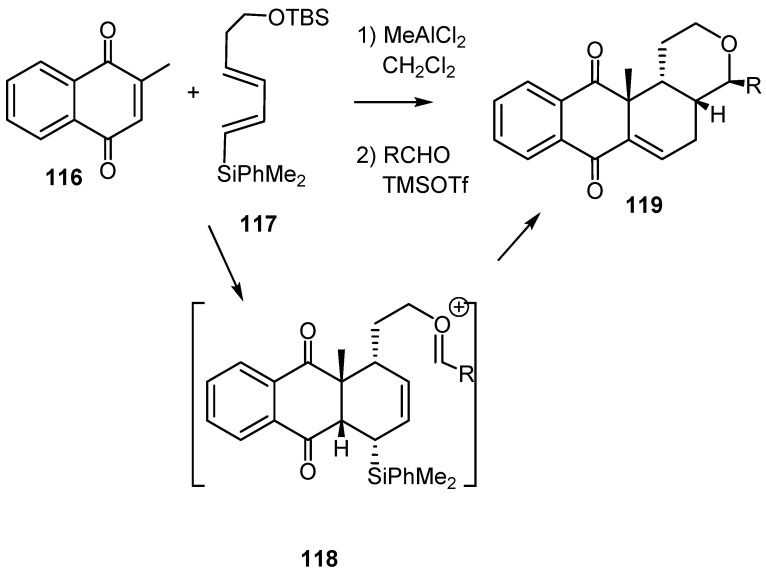
Preparation of isochromenes.

**Scheme 32:**
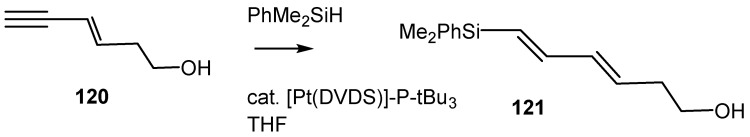
Preparation of a silyl dienol.

Panek and Lee reported the use of a silyl-substituted diene (**117**) in a Diels-Alder reaction with menadione (**116**) as a route to isochromene ring systems (Scheme 31) [30]. The silyl diene used in this chemistry was prepared via a regioselective hydrosilylation of (*E*)-3-hexen-5-yn-1-ol (**120**) using Chandra’s catalyst (Pt(divinyldisiloxane)-P-*t*Bu_3_) (Scheme 32). The silicon-substituted diene (**121**) was then protected and used in a one-pot sequential Diels-Alder/annulation sequence that used MeAlCl_2_ as the catalyst for the Diels-Alder and TMSOTf for the annulation.

**Scheme 33:**
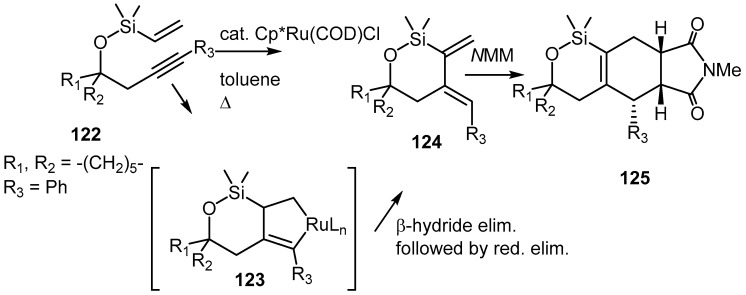
Cycloisomerization of silicon-tethered enynes.

Clark and Kaminsky reported the cycloisomerization of vinyl silicon-tethered 1,7-enynes (**133**) to produce silicon-substituted exocyclic 1,3-dienes (**124**) (Scheme 33) [31]. One of these dienes was then taken on in a Diels-Alder reaction with *N*-methyl maleimide (*N*MM). The optimum catalyst for diene formation via cyclization to a ruthenacyclopentene (**123**) followed by endocyclic β-H elimination was found to be Cp*Ru(COD)Cl. Several procedures for cleavage and functionalization of the silicon tether in these dienes were also reported.

**Scheme 34:**
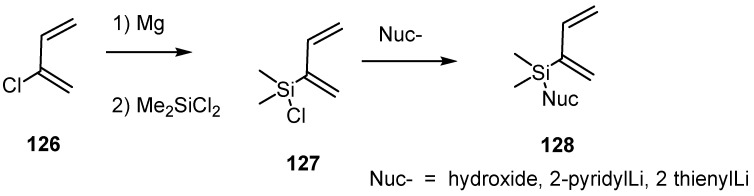
Reactions of chloroprene Grignard reagent with silyl electrophiles.

**Scheme 35:**
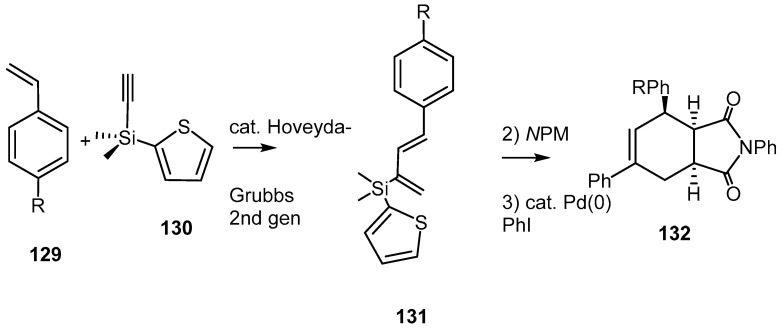
Preparation and reactions of thienyl silyl dienes.

In 2015, we reported synthesis of a number of new silicon-substituted 1,3-butadienes (**128**) via reaction of the Grignard reagent generated from chloroprene (**126**) with silyl electrophiles (Scheme 34) [32]. Diels-Alder reactions with a number of dienophiles were performed and the yields were highest with the 2-thienyldimethylsilyl-substituted diene. Given that, we explored the synthesis of more highly substituted dienes (**131**) within this class via enyne cross metathesis using 2-thienyldimethylsilylethyne (**130**) and substituted styrenes (**129**) (Scheme 35). One-pot cross metathesis/Diels-Alder reactions of these dienes with *N*-phenylmaleimide were then effected with diastereoselectivities >27:1. The resulting silyl-substituted cycloadducts were cross-coupled with iodobenzene to yield **132**.

**Scheme 36:**
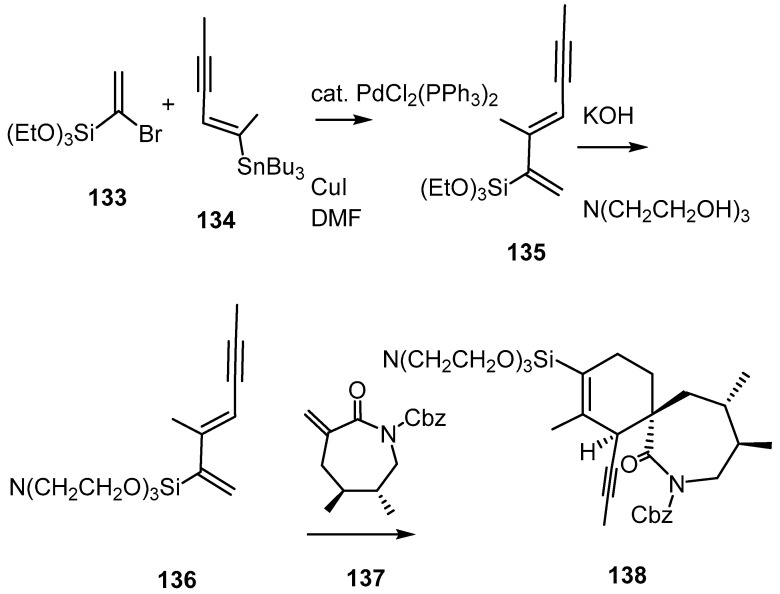
Preparation and reactions of silicon-substituted dienynes.

Murata and co-workers reported the preparation of two silicon-substituted dienynes (**135** and **136**), one of which was used in a highly selective Diels-Alder reaction to prepare the spirocyclic pharmacophore of spirolide C (**138**) (Scheme 36) [33]. The triethoxysilyl-substituted dienyne (**135**) was prepared using a Stille coupling of 1-bromo-1-triethoxysilyl ethene (**133**) with an (*E*)-stannyl enyne (**134**). Treatment of that dienyne (**135**) with KOH and triethanolamine under Dean-Stark conditions produced the 2-silatrane dienyne (**136**). Heating the silatrane dienyne (**136**) with the α-methylene-ε-lactam (**137**) in toluene gave the desired spirocyclic Diels-Alder product (**138**) in a 3:1 ratio over the other stereoisomer in 79% yield.

**Scheme 37:**
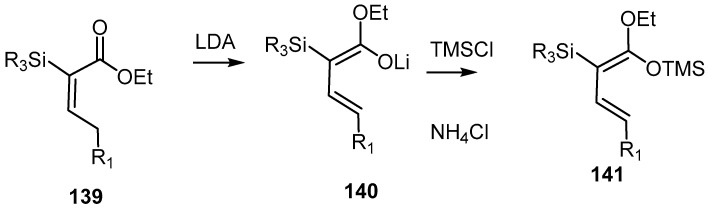
Preparation of silyl ketene acetals.

Johnson, Hendrix and Jennings reported syntheses of a number of silyl ketene acetals (**141**) from deprotonation and TMSCl trapping of α-silyl-α,β-unsaturated esters (**150**) (Scheme 37) [34]. No Diels-Alder reactions of these dienes (**141**) were reported but isolated yields of diene products ranged from 85% to 99% and *E:Z* ratios across both double binds in all products were >20:1.

**Scheme 38:**
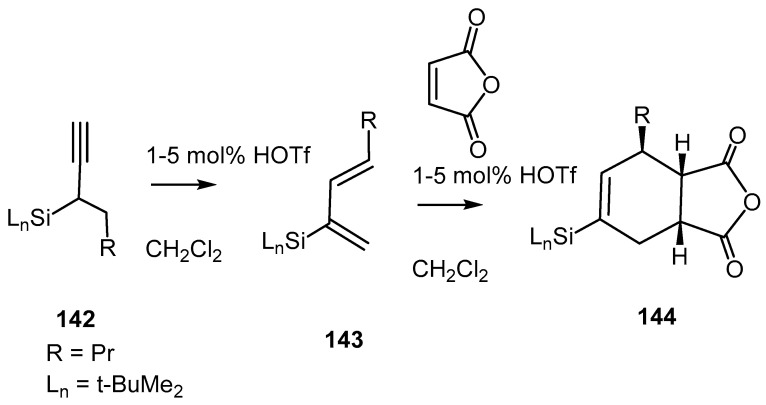
Silyl shift of propargyl silanes.

Turks and co-workers reported an acid-catalyzed 1,2-silyl shift in propargyl silanes (**142**) as a route to silyl-substituted dienes (**143**) (Scheme 38) [35]. A variety of metal triflates were first investigated as catalysts for this reaction but the authors ultimately determined that small amounts of triflic acid sonicated in CH_2_Cl_2_ caused rapid isomerizations in high yields for a variety of silanes. In one case, a *t*-butyldimethylpropargyl silane was treated with 1% HOTf in the presence of maleic anhydride for 3 h at 25 °C and the cycloadduct (**144**) resulting from a Diels-Alder reaction via an exo transition state was isolated in 66% yield.

**Scheme 39:**
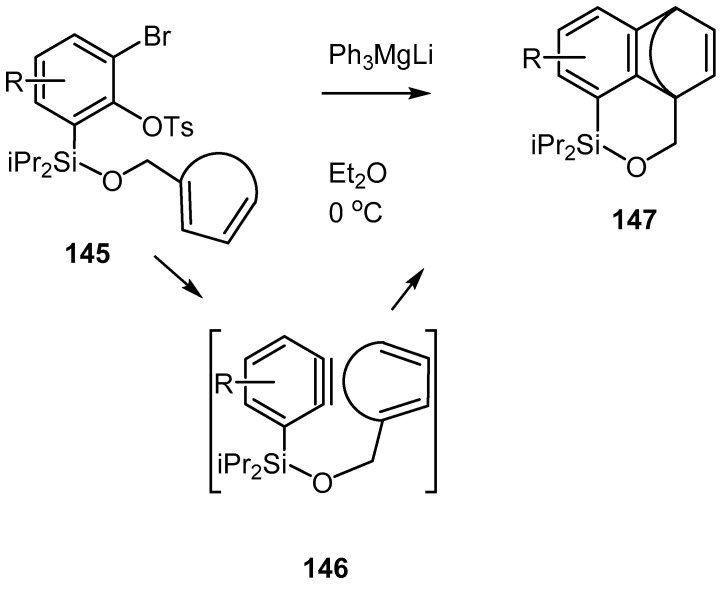
Intramolecular benzyne-diene Diels-Alder reactions.

Lastly, while not technically involving a silyl-substituted diene or dienophile reaction (unless we consider the silyl benzyne as silyl diene + alkyne), Suzuki and co-workers reported using bromosilylphenyl tosylates (**145**) as precursors for intramolecular benzyne-diene Diels-Alder reactions utilizing a silicon tether (**146**) (Scheme 39) [36]. A variety of alkyl lithium and Grignard reagents were investigated as metalating agents but the magnesite (Ph_3_MgLi) provided the highest yields of products (**147**) in a variety of ether solvents. Acyclic *E* dienes and aromatic ring systems also reacted via [4+2] cycloaddition with these benzynes whereas acyclic *Z* dienes reacted via [2+2] cycloaddition.

### 4.2. Siloles

Like boroles, there are many publications on siloles and their uses in photochemical and electrochemical applications. The number of reports on their synthesis and use in cyclization or cycloaddition reactions is much smaller.

**Scheme 40:**
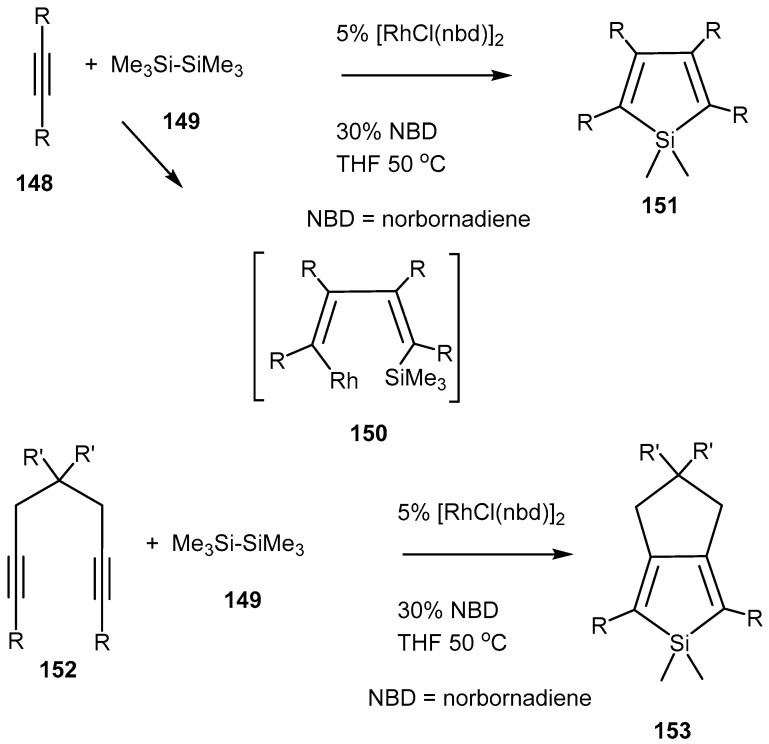
Synthesis of siloles from hexamethyldisilane.

Matsuda and co-workers reported a synthesis of siloles (**151**) using a rhodium-catalyzed inter and intramolecular cyclization of alkynes and diynes with hexamethyldisilane (**149**) (Scheme 40) [37]. This reaction proceeded in 30–70% yields for a number of symmetrical alkynes (**148**) but yielded intractable mixtures or dienes rather than siloles with unsymmetrical alkynes. The reaction was proposed to start with transmetallation to produce a Rh-SiMe_3_ species which does two alkyne insertions to produce intermediate (**150**). Oxidative addition of a SiMe bond followed by reductive elimination would produce the silole (**151**) plus a Rh-Me species that could continue the cycle.

Chang, Kaiser and co-workers reported a gas phase synthesis of 1-silacyclopenta-2,4-diene that was specifically designed to prevent Diels-Alder dimerization of this silole [38]. The silole was prepared via reaction of the silyl radical silylidyne (SiH) with 1,3-butadiene. 

**Scheme 41:**
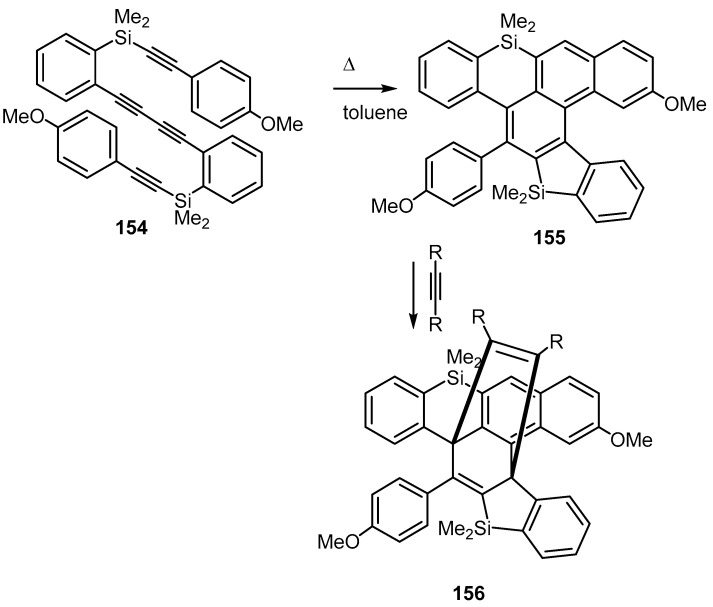
Reactions of silicon tethered tetraynes.

Shibata and co-workers reported consecutive hexadehydro-Diels-Alder (HDDA) and tetradehydro-Diels-Alder (TDDA) reactions of silicon tethered tetraynes (**154**) that produced dibenzosilole-fused polycyclic products (**155**) (Scheme 41) [39]. The reaction proceeded for a variety of dimethylsilyl and diphenylsilyl aromatic tetraynes in yields from 43–81%. One of the silahelicene products (**155**) of these HDDA/TDDA sequences was then taken on as a silyl diene component in Diels-Alder reactions with benzyne and dimethyl acetylenedicarboxylate to yield **156** (R = CO_2_Me).

## 5. Silicon Dienophiles

**Scheme 42:**
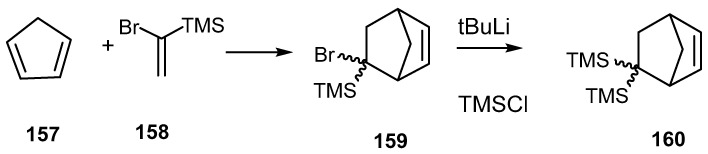
Preparation of bicycloheptene diyl bis(trimethylsilane).

Westiuk and Sokol reported a DFT computational study on several possible routes to prepare bicyclo [2.2.1]hept-5-ene-2,2-diylbis(trimethylsilane) (**160**) by Diels-Alder reactions of cyclopentadiene (**157**) and silyl dienophiles (**158**) (Scheme 42) [40]. The reaction with the most favorable ΔG for the forward reaction (~10 kcal/mol) and the most unfavorable ΔG for the retro Diels-Alder reaction (~41 kcal/mol) used (1-bromovinyl)trimethylsilane (**158**) so this was the route they followed to prepare the target cycloadduct (**160**).

**Scheme 43:**
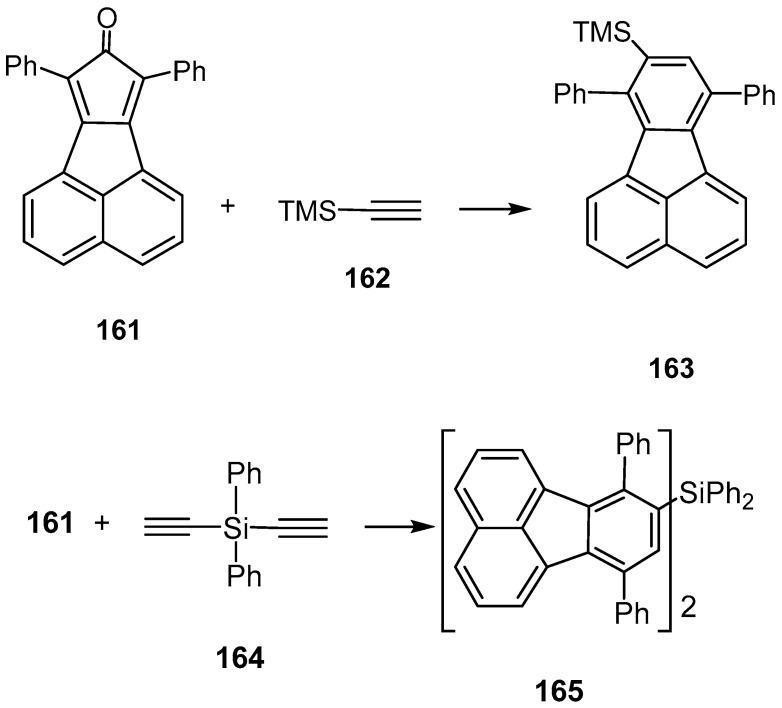
Preparation of fluoranthenes.

Feng and co-workers used ethynyl-trimethylsilane (**162**) and diethynyldiphenylsilane (**164**) as dienophiles in Diels-Alder approaches to fluoranthenes (**163** and **165**) which were used as fluorescent probes for detecting nitroaromatic compounds (Scheme 43) [41]. The ethynyl-trimethylsilane (**162**) underwent Diels-Alder reaction with a diphenyl-cyclopentaacenaphthylen-8-one (**172**) in 55% yield and the diethynyldiphenylsilane (**164**) reacted with this same diene in 30% yield.

**Scheme 44:**
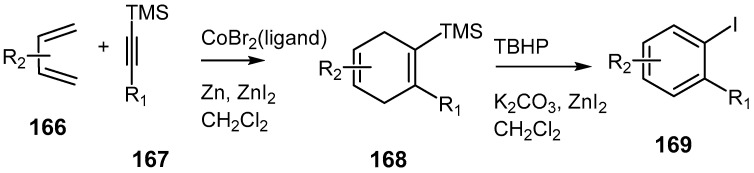
Diels-Alder reactions of alkynyl silanes.

Hilt and Mockel reported the preparation of polysubstituted iodobenzene derivatives (**169**) via Diels-Alder/oxidation/iodination sequences starting from aryl dienes (**166**) and alkynyl silanes (**167**) (Scheme 44) [42]. This group had previously reported conditions for cobalt-catalyzed Diels-Alder reactions of simple dienes with alkynyl silanes and this study focused more on optimizing reaction conditions for the oxidation/iodination sequence that followed the Diels-Alder reaction. The optimized oxidation/iodination protocol involved using *t*-butylhydroperoxide (TBHP) as an oxidant under basic conditions with ZnI_2_ as the iodine source.

**Scheme 45:**
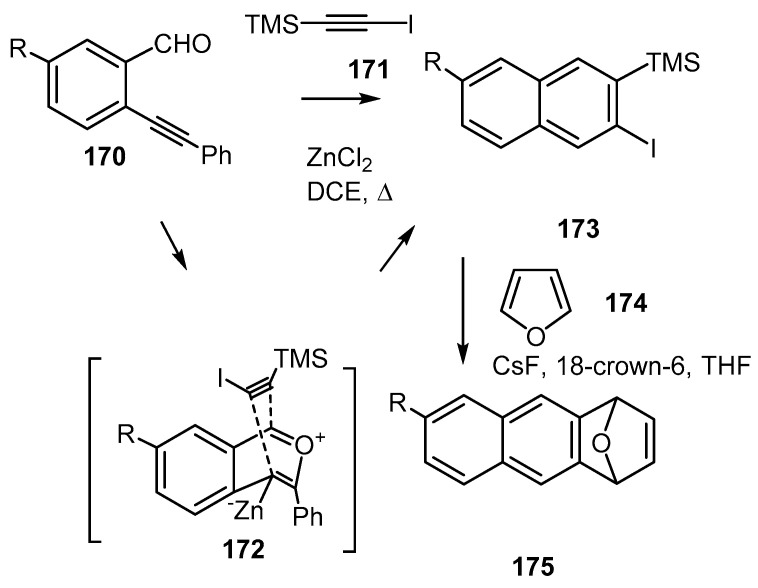
Preparation of silicon-substituted naphthyne precursors.

Lastly in this section on silicon dienophiles, Dichtel and co-workers reported preparation of silyl-substituted naphthalenes via benzannulation of halogenated silyl alkynes (**171**) (Scheme 45) [43]. Following that initial silyl alkyne Diels-Alder sequence, the silyl iodonaphthalenes (**173**) produced were then treated with CsF in a number of cases to produce benzynes that were trapped via Diels-Alder reactions with furan (**174**).

## 6. Conclusions

Boron and silicon substituted dienes and dienophiles continue to be heavily studied. Their ease of synthesis and handling coupled with their ability to serve as surrogates for a variety of other functional groups via subsequent cross coupling, oxidation, or halogenation chemistry is responsible for their ongoing study and use by synthetic chemists. As new, milder and more functional group tolerant reaction conditions continue to be explored for cross coupling reactions, one would predict that utilization of main group element substituted dienes and dienophiles will become even more widespread in the future.
